# Postoperative *Tropheryma whipplei* endophthalmitis – a case report highlighting the additive value of molecular testing

**DOI:** 10.1099/jmmcr.0.005124

**Published:** 2017-10-23

**Authors:** Julia Dick, Patrizia Krauß, Jost Hillenkamp, Britta Kohlmorgen, Christoph Schoen

**Affiliations:** ^1^​ University of Würzburg, Institute for Hygiene and Microbiology, Josef-Schneider-Str. 2 E1, 97080 Wuerzburg, Germany; ^2^​ Department of Ophthalmology, University Hospital Wuerzburg, Josef-Schneider-Str. 11, 97080 Wuerzburg, Germany

**Keywords:** Whipple's disease, endophthalmitis, *Tropheryma whipplei*, ocular infection, vitrectomy, intravitreal vancomycin and amikacin, intravenous ceftriaxone, oral cefpodoxime, topic ofloxacin, oral doxycycline

## Abstract

**Introduction.** *Tropheryma whipplei* is the causative agent of Whipple’s disease. Gastrointestinal and lymphatic tissues are affected in the majority of cases, resulting in diarrhoea, malabsorption and fever. Here, we report a rare case of ocular manifestation in a patient lacking the typical Whipple symptoms.

**Case presentation.** A 74-year-old Caucasian female presented with blurred vision in the right eye over a period of 1–2 months, accompanied by stinging pain and conjunctival hyperaemia for the last 2 days. Upon admission, visual acuity was hand motion in the affected eye. Ophthalmological examination showed typical signs of intraocular inflammation. Diagnostic and therapeutic pars plana vitrectomy including vitreous biopsy and intravitreal instillation of vancomycin and amikacin was performed within hours of initial presentation. Both microscopic analysis and microbial cultures of the vitreous biopsy remained negative for bacteria and fungi. The postoperative antibiotic regime included intravenous administration of ceftriaxone in combination with topical tobramycin and ofloxacin. Due to the empirical therapy the inflammation ceased and the patient was discharged after 5 days with cefpodoxime orally and local antibiotic and steroidal therapy. Meanwhile, the vitreous body had undergone testing by PCR for the eubacterial 16S rRNA gene, which was found to be positive. Analysis of the PCR product revealed a specific sequence of *T. whipplei*.

**Conclusion.** In our patient, endophthalmitis was the first and only symptom of Morbus Whipple, while most patients with Whipple’s disease suffer from severe gastrointestinal symptoms. 16S rDNA PCR should be considered for any intraocular infection when microscopy and standard culture methods remain negative.

## Abbreviation

WD, Whipple’s disease.

## Introduction

*Tropheryma whipplei* is a bacterium of the phylum *Actinobacteria* and causes Whipple’s disease (WD) [1]. The rod-shaped bacterium can be visualized by electron microscopy; diagnostic identification by light microscopy fails, as *T. whipplei* cannot be stained with conventional dyes. Culturing is difficult; the first stable cultures of *T. whipplei* were established only in 2000 [2]. Standard culturing methods used in diagnostic bacteriology laboratories do not provide sufficient substrate for this fastidious bacillus.

WD was first described in 1907 by George H. Whipple and was suspected to be an infectious disease [3]. Although pathognomonic periodic acid-Schiff (PAS) inclusions in the cytoplasm of macrophages were visible in the majority of tissue samples from Whipple patients, the causative bacterium was not identified until 1992 [4].

WD is characterized by diarrhoea, abdominal pain and arthralgia [5]. Weight loss and fever are common, which may be misinterpreted as B-symptoms. Epidemiological data are difficult to obtain for the disease; estimates on the basis of indirect evidence suggest a prevalence of about 1 : 1 000 000 and an annual incidence of one to six per 10 million inhabitants [6]. Because of the rarity of the disease, controlled randomized therapeutic trials are difficult. Therefore, antibiotic treatment strategies are often derived from long-term follow-up case reports [7].

Among WD patients, ocular involvement is rare and is usually a late manifestation. The dimension of ocular WD is heterogeneous, and can be found as uveitis, chorioiditis, retinitis, keratitis and endophthalmitis as well as neuro-ophthalmological disease, often correlated with central nervous system infection [8]. Most patients with eye involvement state gastrointestinal symptoms or have been previously diagnosed with WD [9]. Here, we report a rare case of postoperative endophthalmitis in a patient lacking the typical Whipple symptoms.

## Case report

A 74-year-old Caucasian female presented with a 1–2 month history of blurred vision of the right eye, accompanied by stinging pain and conjunctival hyperaemia for the last 2 days. The patient had undergone cataract surgery on the left eye 7 months earlier and on the affected right eye 4 months earlier. At time of admission she was treated with cortisone eye drops due to a purulent conjunctivitis that developed about 1 week prior to admission. A urinary tract infection had been treated with ciprofloxacin until 2 days before admission. General history included chronic lymphatic leukaemia for a period of 30 years, Parkinson’s disease, status post-melanoma and post-hip replacement surgery. The patient was allergic to cotrimoxazole.

Upon admission, visual acuity was hand motion in the right eye and 0.4 (decimal acuity) in the left eye, and intraocular pressures were 14 mmHg in the right eye and 16 mmHg in the left eye. Ophthalmological examination of the right eye showed Descemet membrane folds, hypopyon, anterior chamber inflammation, endothelial cells and fibrin coating on the back face of the intraocular lens. The vitreous body showed inflammatory cells and streaks. Fundoscopy revealed a whitish retinal lesion with blurred margins in the lower midperiphery. The left eye did not show any signs of intraocular inflammation.

The patient was presumptively diagnosed with acute endophthalmitis of the right eye.

## Investigations

Within a few hours of admission, the patient underwent vitrectomy including vitreous biopsy. The specimen was examined microscopically and microbial culturing was initiated immediately. Culturing included plating on Columbia agar with 5 % sheep blood (bioMérieux) and chocolate agar [Becton Dickinson (BD)], and liquid culturing in thioglycollate medium (BD) and Sabouraud broth with 4 % glucose (in-house product).

**Fig. 1.**
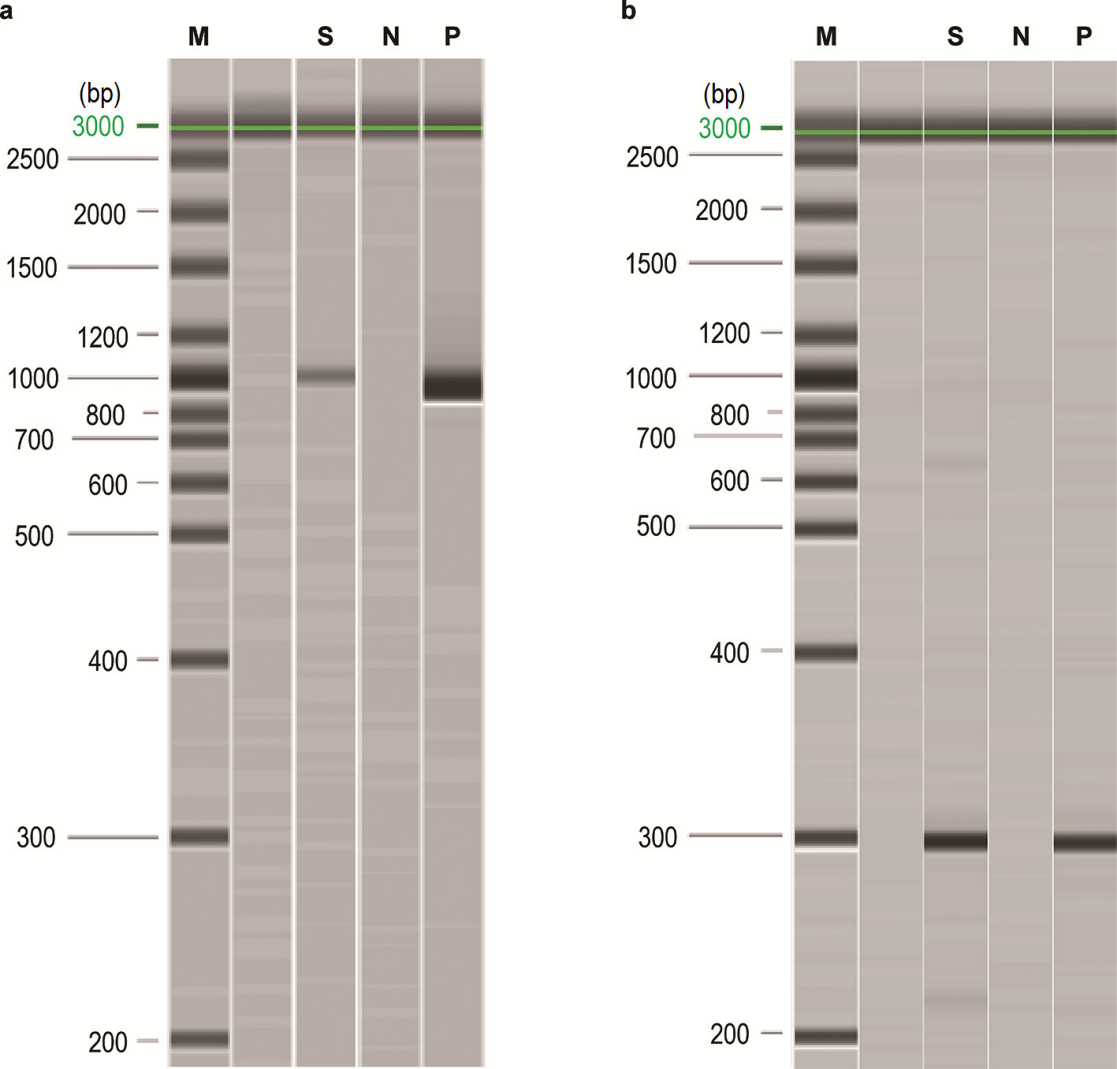
Products of (a) 16S rRNA PCR and (b) *T. whipple* *i*-specific PCR, documented with the QIAxcel ScreenGel system (Qiagen). The size of the amplicon from the eubacterial 16S rRNA PCR is about 800 bp, which is somewhat species-dependent. In the *T. whipplei*-specific PCR, the anticipated size is 284 bp. M: marker with band sizes as indicated, N: negative control, P: positive control, S: patient sample.

In total, 200 µl was retained and stored at 4 °C for molecular testing on the following working day. Microscopic examination of the vitreous body showed neither bacteria nor fungi in the Gram stain. Microbial cultures of the vitreous biopsy remained negative for 12 days. Meanwhile, the vitreous body had undergone testing by PCR for the eubacterial 16S rRNA gene (Fig. 1a), which was found to be positive (primers: BAK 5′-AGTTTGATCHTGGCTCAG-3′ and PC3mod 5′-GGACTACHAGGGTATCTAAT-3’; anticipated amplicon size about 800 bp [10, 11]). The result was documented with a QIAxcel ScreenGel system (Qiagen), and the amplicon was subjected to Sanger sequencing with subsequent alignment against five databases: NCBI nucleotide blast, leBIBI, SepsiTest, Greengenes and RDP_II [12–16].

Surprisingly, sequence analysis of the product indentified *T. whipplei*, which was confirmed by a positive *T. whipplei*-specific PCR (primers: pW3FE 5′-GGAATTCCAGAGATACGCCCCCCGCAA-3′ and pW2RB 5′-CGGGATCCCATTCGCTCCACCTTGCGA-3′; anticipated amplicon size 284 bp [4]) (Fig. 1b).

## Diagnosis

Clinical examination along with molecular testing thus confirmed the diagnosis of postoperative endophthalmitis caused by *T. whipplei*.

## Treatment

Diagnostic and therapeutic pars plana vitrectomy including surgical capsulotomy, lavage of the anterior chamber and intravitreal instillation of vancomycin and amikacin were performed within hours of initial presentation.

The postoperative antibiotic regime included intravenous administration of ceftriaxone in combination with hourly topical tobramycin and ofloxacin. Prednisolone acetate eye drops four times per day and cyclopentolate eye drops twice per day were applied as supportive local therapy. Microbial cultures of the vitreous biopsy remained negative for 12 days. Screening for a source of infection included urine examination, which was sterile but positive for erythrocytes and leucocytes consistent with the pre-existing cystitis. X-ray of the left hip showed no focus of infection. Due to the empirical therapy the inflammation ceased and the patient was discharged after 5 days with oralized antibiotic admission of cefpodoxime 200 mg twice a day and local antibiotic and steroidal therapy. Decimal visual acuity on the right eye had improved to 0.5 on discharge.

## Outcome and follow-up

Four weeks later the patient presented for follow-up. Decimal visual acuity then was 0.8 in the right eye and 0.7 in the left eye; intraocular pressures were 13 and 14 mmHg. No intraocular inflammation was detectable, so local therapy was stopped.

During follow-up care the patient was informed about the pathogen found in the vitreous fluid, and about WD. The consequences, such as the importance of further diagnostic procedures such as lumbar puncture for cerebrospinal fluid examination and duodenoscopy to exclude multi-organ involvement of WD and the schedule for antibiotic treatment, were discussed together with the patient, her immediate family and physicians from the Department of Ophthalmology and the Institute for Hygiene and Microbiology. The patient declined further invasive diagnostic treatment because of her pre-existing morbidity; however, she agreed to empirical antibiotic treatment. In consent with the patient, we recommended a long-term-antibiotic oral therapy for treatment of WD without further diagnostic investigation. Due to the patient’s allergy to cotrimoxazole, doxycycline treatment was scheduled with 100 mg twice per day for at least 1 year.

## Discussion

In this report, we describe a rare case of postoperative eye infection in which *T. whipplei* was identified as the causative agent. Empirical treatment with instillation of vancomycin and amikacin plus topical ofloxacin, supported by intravenous ceftriaxone and oral cefpodoxime, respectively, led to significant improvement of the patient’s symptoms before the pathogen could be identified. Eye infections after eye surgery or intravitreal injections performed in an operating room are commonly caused by coagulase-negative staphylococci and other Gram-positive bacteria [17, 18]. Postoperative endophthalmitis caused by *T. whipplei* is extremely rare. On describing the first case of postoperative ocular WD in 2008, Drancourt *et al.* retrospectively analysed cases of Whipple eye infections [19]. Among 19 patients, 11 had undergone eye surgery prior to diagnosis of Whipple uveitis [20]. Similar to our patient, they had received topical steroids in the context of cataract surgery and then developed symptoms of chronic postoperative pan-endophthalmitis. Other clinical manifestations of eye involvement in WD were described earlier, such as vitritis, (chorio-)retinitis, (pan-)uveitis, papilloedema, optical atrophy, retinal haemorrhage and crystalline keratopathy [21–29].

Chiquet *et al.* showed that identification of bacteria in endophthalmitis patients by culturing may lead to false-negative results, especially when the patient has received antibiotics before the sample is drawn, and concluded that eubacterial PCR should be performed complementary to standard microbial culturing [30]. This procedure is also recommended by the guidelines on endophthalmitis following cataract surgery of the European Society of Cataract and Refractive Surgery [31].

Evaluation of data obtained in the diagnostic laboratory of the Institute for Hygiene and Microbiology at the University of Würzburg over a 3-year period (2014–2016) showed 46 cases of suspected bacterial endophthalmitis that had been examined via conventional microbial culture and 16S rDNA PCR plus sequencing as described above. Twenty-one vitreous body aspirates, 10 aqueous humour samples and 15 aspirates of undefined origin were included. In 22 cases, cultures remained sterile; among material from these 22 cases, four were detected as positive by 16S rDNA PCR. Thus, 18 % of all specimens without evidence of microbial growth were found to give false-negative results. Sequence analysis of the 16S rRNA gene revealed viridians streptococci, *Moraxella nonliquefaciens*, *T. whipplei* and one polymicrobial infection. Identification was based on sequence alignment with five different databases: NCBI nucleotide blast, leBIBI, SepsiTest, Greengenes and RDP_II [12–16].

Fastidious bacteria were exclusively identified by PCR, while Gram-positive cocci as the most common pathogens in postoperative eye infections could be diagnosed by culture and verified by PCR. A possible explanation for the positive PCR and negative culture results is the loss of bacterial viability, which may occur if the sample transportation exceeds the critical time limit for fastidious bacteria [32, 33]. Nonetheless, bacterial DNA is still present in these samples and can be detected by PCR.

In three cases, cultures showed growth of *Staphylococcus epidermidis* or *Propionibacterium acnes*, while the native aspirate tested negative by PCR. This might be due to the fact that the sensitivity of the 16S rDNA PCR employed was limited to 100 genome equivalents in aqueous solution to reduce the number of false-positive results caused by spurious amounts of pervasive bacterial DNA. In contrast, microbial culture is significantly more sensitive for non-fastidious bacteria. Another explanation might be the degradation of bacterial DNA during storage, as PCR is routinely performed only during weekdays at our microbiology department and samples are meanwhile kept at 4 °C [34, 35]. However, we cannot exclude the possibility that these cultures might have been contaminated and the PCR had therefore remained sterile (Table 1).

**Table 1.** Bacteria identified from vitreous or aqueous humour samples of patients with diagnosed endophthalmitis over a 3-year period (2014–2016)

**Result**	**Total**	**PCR**	**Culture**	**Both**
Positive	27	23	22	18
*Staphylococcus epidermidis*	11	9	11	9
*Staphylococcus aureus*	2	2	2	2
Oral streptococci	5	5	4	4
*Streptococcus pneumoniae*/*pseudopneumoniae*	2	2	2	2
*Serratia marscescens*	1	1	1	1
*Propionibacterium acnes*	1	0	1	0
*Tropheryma whipplei*	1	1	0	0
*Moraxella nonliquefaciens*	1	1	0	0
Other	2	1	1	0
Not distinguishable (PCR≥2 species)	1	1	0	0
Negative	19	23	24	28

### Conclusion

This report shows how standard microbiological culturing may fail to identify the causative agent of acute endophthalmitis and emphasizes the importance of complementary methods, such as 16S rDNA PCR. Postoperative eye infections due to *T. whipplei* are extremely rare; nonetheless, 16S rDNA PCR should be routinely performed on vitreous samples additional to standard microbial culturing to identify fastidious bacteria.

## References

[1] Ventura M, Canchaya C, Tauch A, Chandra G, Fitzgerald GF (2007). Genomics of Actinobacteria: tracing the evolutionary history of an ancient phylum. Microbiol Mol Biol Rev.

[2] La Scola B, Fenollar F, Fournier PE, Altwegg M, Mallet MN (2001). Description of *Tropheryma whipplei* gen. nov., sp. nov., the Whipple's disease bacillus. Int J Syst Evol Microbiol.

[3] Whipple GH (1907). A hitherto undescribed disease characterized anatomically by deposits of fat and fatty acids in the intestinal and mesenteric lymphatic tissues. Bull Johns Hopkins Hosp.

[4] Relman DA, Schmidt TM, Macdermott RP, Falkow S (1992). Identification of the uncultured bacillus of Whipple's disease. N Engl J Med.

[5] Marth T, Raoult D (2003). Whipple's disease. Lancet.

[6] von Herbay A, Otto HF, Stolte M, Borchard F, Kirchner T (1997). Epidemiology of Whipple's disease in Germany. Analysis of 110 patients diagnosed in 1965–95. Scand J Gastroenterol.

[7] Marth T, Moos V, Müller C, Biagi F, Schneider T (2016). *Tropheryma whipplei* infection and Whipple's disease. Lancet Infect Dis.

[8] Chan RY, Yannuzzi LA, Foster CS (2001). Ocular Whipple's disease: earlier definitive diagnosis. Ophthalmology.

[9] Touitou V, Fenollar F, Cassoux N, Merle-Beral H, Lehoang P (2012). Ocular Whipple's disease: therapeutic strategy and long-term follow-up. Ophthalmology.

[10] Wilson KH, Blitchington RB, Greene RC (1990). Amplification of bacterial 16S ribosomal DNA with polymerase chain reaction. J Clin Microbiol.

[11] Goldenberger D, Künzli A, Vogt P, Zbinden R, Altwegg M (1997). Molecular diagnosis of bacterial endocarditis by broad-range PCR amplification and direct sequencing. J Clin Microbiol.

[12] NCBI (2015). Nucleotide BLAST: National Center for Biotechnology Information. https://blast.ncbi.nlm.nih.gov/Blast.cgi?PAGE_TYPE=BlastSearch.

[13] leBIBI (2015). Prokaryotes Phylogeny Facility - PPF: Laboratoire de Biométrie et Biologie Évolutive. https://umr5558-bibiserv.univ-lyon1.fr/lebibi/lebibi.cgi.

[14] SepsiTest (2015). BLAST: Molzym Molecular Diagnostics. http://www.sepsitest-blast.de/en/index.html.

[15] greengenes (2015). a Chimera-Checked 16S rRNA Gene Database: Lawrence Berkeley National Laboratory. http://greengenes.lbl.gov/Download/.

[16] Cole JR, Wang Q, Cardenas E, Fish J, Chai B (2009). The ribosomal database project: improved alignments and new tools for rRNA analysis. Nucleic Acids Res.

[17] Ng JQ, Morlet N, Pearman JW, Constable IJ, McAllister IL (2005). Management and outcomes of postoperative endophthalmitis since the endophthalmitis vitrectomy study: the endophthalmitis population study of western Australia (EPSWA)'s fifth report. Ophthalmology.

[18] Dossarps D, Bron AM, Koehrer P, Aho-Glélé LS, Creuzot-Garcher C (2015). Endophthalmitis after intravitreal injections: incidence, presentation, management, and visual outcome. Am J Ophthalmol.

[19] Drancourt M, Berger P, Terrada C, Bodaghi B, Conrath J (2008). High prevalence of fastidious bacteria in 1520 cases of uveitis of unknown etiology. Medicine.

[20] Drancourt M, Fenollar F, Denis D, Raoult D (2009). Postoperative panophthalmitis caused by Whipple disease. Emerg Infect Dis.

[21] Rickman LS, Freeman WR, Green WR, Feldman ST, Sullivan J (1995). Brief report: uveitis caused by *Tropheryma whippelii* (Whipple's bacillus). N Engl J Med.

[22] Williams JG (1998). Ocular Manifestations of Whipple disease. Arch Ophthalmol.

[23] Conly JM, Johnston BL (2001). Rare but not so rare: the evolving spectrum of Whipple's disease. Can J Infect Dis.

[24] Drancourt M, Raoult D, Lépidi H, Fénollar F, Birg ML (2003). Culture of *Tropheryma whippelii* from the vitreous fluid of a patient presenting with unilateral uveitis. Ann Intern Med.

[25] Nubourgh I, Vandergheynst F, Lefebvre P, Lemy A, Dumarey N (2008). An atypical case of Whipple's disease: case report and review of the literature. Acta Clin Belg.

[26] Razonable RR, Pulido JS, Deziel PJ, Dev S, Salomão DR (2008). Chorioretinitis and vitreitis due to *Tropheryma whipplei* after transplantation: case report and review. Transpl Infect Dis.

[27] Scheurer RA, Kosmorsky GS, Hoffman GS, Farver C, Lee MS (2010). Can't hear, can't see, and too sore to play. Surv Ophthalmol.

[28] Schoenberger SD, Thinda S, Kim SJ (2012). *Tropheryma whipplei* crystalline keratopathy: report of a case and updated review of the literature. Case Rep Ophthalmol Med.

[29] Gaïni S, Bruun NE, Kollslíð R, Thomsen H, Á Steig T (2014). Whipple's disease involving the eye, the brain, the heart and the gut diagnosed through the eye. Acta Ophthalmol.

[30] Chiquet C, Cornut PL, Benito Y, Thuret G, Maurin M (2008). Eubacterial PCR for bacterial detection and identification in 100 acute postcataract surgery endophthalmitis. Invest Ophthalmol Vis Sci.

[31] European Society of Cataract and Refractive Surgery (2013). ESCRS guidelines for prevention and treatment of endophthalmitis following cataract surgery: data, dilemmas and conclusions. www.escrs.org/downloads/Endophthalmitis-Guidelines.pdf.

[32] Atkins KL, Atkinson RM, Shanks A, Parvin CA, Dunne WM (2006). Evaluation of polymerase chain reaction for group B streptococcus detection using an improved culture method. Obstet Gynecol.

[33] Baron EJ, Miller JM, Weinstein MP, Richter SS, Gilligan PH (2013). A guide to utilization of the microbiology laboratory for diagnosis of infectious diseases: 2013 recommendations by the Infectious Diseases Society of America (IDSA) and the American Society for Microbiology (ASM)(a). Clin Infect Dis.

[34] Schrader C, Schielke A, Ellerbroek L, Johne R (2012). PCR inhibitors - occurrence, properties and removal. J Appl Microbiol.

[35] Jayasudha R, Narendran V, Manikandan P, Prabagaran SR (2014). Identification of polybacterial communities in patients with postoperative, posttraumatic, and endogenous endophthalmitis through 16S rRNA gene libraries. J Clin Microbiol.

